# Lumasiran at birth changes the trajectory of primary hyperoxaluria type 1: same disease, different outcomes in two affected siblings

**DOI:** 10.1007/s40620-025-02325-2

**Published:** 2025-07-09

**Authors:** Licia Peruzzi, Marta Leporati

**Affiliations:** 1 Pediatric Nephrology Unit, Regina Margherita Children’s Hospital, AOU Città della Salute e Della Scienza di Torino, Piazza Polonia 94, 10126 Turin, Italy; 2 European Reference Network for Rare Kidney Diseases (ERKNet) Center, Turin, Italy; 3 Analytical Chemistry and Kidney Stone, Laboratory, AO Ordine Mauriziano, Turin, Italy

**Keywords:** Primary hyperoxaluria type 1, RNA interference, Lumasiran, Nephrocalcinosis

## Abstract

Lumasiran, an RNA interference therapeutic, demonstrated effectiveness in clinical trials, leading to approval for primary hyperoxaluria type 1 management in all age groups. To date, little is known about its use in newborns. This study assesses, for the first time, the oxalate and glycolate metabolism in a newborn affected by primary hyperoxaluria type 1 treated at birth. His older brother, also affected by primary hyperoxaluria type 1, experienced severe disease progression and significant comorbidities. These challenges informed the decision to initiate immediate treatment for the younger sibling. The child was treated at 6 h of life with lumasiran 6 mg/kg subcutaneously, in combination with pyridoxin 10 mg/kg/day. Lumasiran 6 mg/kg was repeated at 30 and 60 days, then was reduced to 3 mg/kg every month. Intravenous hyperhydration (240 mL/kg/day) was maintained for 16 days, together with oral water and potassium citrate (500 mg in 500 mL/day) in addition to breastfeeding. Although gycolate oxidase inhibition was started immediately after birth in the absence of previous deposits, it showed a latency of at least 15 days. Over this period of time, dangerous levels of blood and urinary oxalate were reached, due to the physiological low glomerular filtration rate in the perinatal period, as demonstrated by the increasing levels of endogenous oxalate production until day 6. Blood oxalate supersaturation 30 days after the first dose of treatment was never reached again. No adverse events occurred. In this report, early treatment with lumasiran, coupled with hyperhydration and supportive therapy, was able to ensure the absence of primary hyperoxaluria type 1 symptoms throughout the 24 months of follow-up.

**Graphical abstract:**

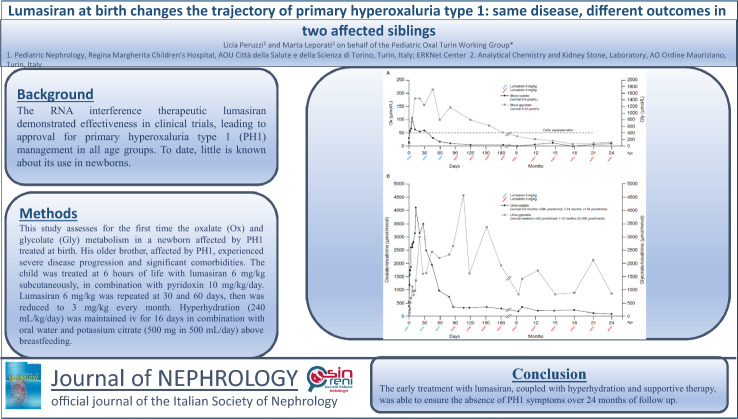

**Supplementary Information:**

The online version contains supplementary material available at 10.1007/s40620-025-02325-2.

## Introduction

Primary hyperoxaluria type 1 is a rare autosomal recessive disease resulting from mutations in the alanine-glyoxylate aminotransferase *AGXT* gene [1, 2]. The consequent deficiency of the liver-specific peroxisomal enzyme alanine-glyoxylate aminotransferase (AGT) results in increased glyoxylate and oxalate production and excessive urinary oxalate excretion, causing the formation of kidney stones and oxalate-induced crystal nephropathy or nephrocalcinosis due to the poor urinary calcium oxalate solubility [3]. Broadly, traditional management involves symptomatic treatment for the less severe forms, consisting of intensive hyperhydration, potassium citrate supplementation and conservative chronic kidney disease management [4, 5]. Vitamin B6 (pyridoxin) can be useful for patients with specific variants [6]. Intensive hemodialysis should be performed in patients with kidney failure to avoid blood calcium oxalate supersaturation and irreversible systemic oxalosis.^5^ In the most severe forms, a combined liver and kidney transplant is required. In contrast, kidney transplantation alone leads to disease recurrence and graft loss [7]. The patients’ quality of life is highly affected by such challenging procedures, and significant associated morbidity and mortality are reported [8, 9].

Lumasiran, an RNA interference therapeutic, is a glycolate oxidase-targeting agent that blocks the enzyme cascade upstream, inducing the accumulation of glycolate instead of oxalate [10]. Lumasiran demonstrated both effectiveness and tolerability in clinical trials, leading to approval by the US Food and Drug Administration (FDA) and the European Medicines Agency (EMA) for use in primary hyperoxaluria type 1 in all age groups [11–14].

To date, fewer than 400 patients are being treated with lumasiran worldwide, and little is known about its use in infants and newborns [13–15]. This study presents the first 24-month assessment of oxalate and glycolate metabolism in a newborn affected by primary hyperoxaluria type 1 who was treated with lumasiran, an RNA-interfering drug, starting 6 h after birth, which proved completely effective in preventing the onset of the disease.

## Case report

### Patient history

Prenatal diagnosis of primary hyperoxaluria type 1 (homozygous AGXT c.731T>C9 [p.(Ile244Thr)] mutation) was made at 11 weeks of pregnancy through chorionic villus sampling performed due to family history.

The parents, who were first cousins and heterozygous carriers, had had a first child affected by primary hyperoxaluria type 1 five years earlier, who reached kidney failure in the second month of life, requiring intensive hemodialysis. Liver and kidney transplantation was performed at 13 months of age, however after one month the kidney graft was lost due to disease recurrence presumably to the mobilization of the systemic oxalate deposits. Multiple comorbidities occurred, a second kidney transplant was required and the child developed post-transplant lymphoproliferative disease.

The second pregnancy was closely monitored by a multidisciplinary team; maternal blood and urine oxalate and glycolate levels were normal. The fetus, large for gestational age, due to gestational diabetes, had normal morphology. A cesarean section at 37 weeks resulted in the birth of a 4120 g male infant, Apgar score 9/9. The child underwent serial blood and urine oxalate measurements starting from the cord blood and amniotic fluid, which highlighted a rapid increase in oxalate in the first hours.

At 6 h of life, to prevent nephrocalcinosis development, the child was treated with glycolate oxidase RNA interference lumasiran 6 mg/kg subcutaneously, associated with pyridoxin 10 mg/kg/day. Sanger sequencing confirmed both the homozygous mutation *AGXT* c.731T>C9 [p.(Ile244Thr)], which is only partially responsive to vitamin B6, and the diagnosis of primary hyperoxaluria type 1.

Intravenous hyperhydration (240 mL/kg/day) was maintained for 16 days, together with oral water and potassium citrate (500 mg in 500 mL/day) in addition to breastfeeding.

Blood and urine oxalate and glycolate were assessed every 48 h for the first week, then every ten days and subsequently before each administration of lumasiran, using ion chromatography and liquid chromatography-tandem mass spectrometry, respectively. Certified standard solutions were used for calibration. Nine-point calibration curves with internal standard (glycolic acid ^13^C_2_) were used for the determination of plasma and urine glycolate. A single point calibration curve with internal standard (bromide) was used for the determination of plasma and urine oxalate. Urinary creatinine was determined using an enzymatic colorimetric method. Liver and kidney function, acid–base parameters and electrolytes were assessed before each lumasiran administration.

Lumasiran 6 mg/kg was repeated at 30 and 60 days, then the dose was reduced to 3 mg/kg every month, according to schedule and to 6 mg/kg every 90 days after the patient reached a weight of 10 kg.

### Treatment outcomes

Cord blood oxalate was 15 μmol/L (normal < 10 μmol/L), and amniotic fluid oxalate and glycolate were 55 μmol/L (normal 19–71 μmol/L) and 2 μmol/L (normal 66–109 µmol/L), respectively. The first urine test showed an oxalate/creatinine ratio of 401 µmol/mmol (normal < 360 µmol/mmol) and a glycolate/creatinine (UGly/Cr) ratio of 6 µmol/mmol (normal < 50 µmol/mmol).

**Fig. 1:**
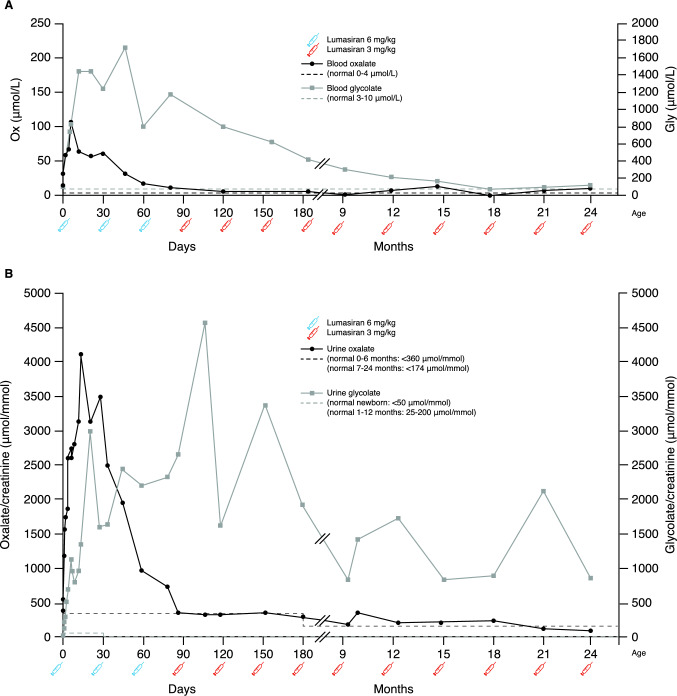
Oxalate and glycolate concentrations in a newborn affected by primary hyperoxaluria type 1 treated with lumasiran starting from 6 h of life. **A** Blood oxalate and glycolate concentrations. **B** Spot urine oxalate and glycolate to creatinine. *Ox* oxalate, *CaOx* calcium oxalate, *Gly* glycolate

At 6 h of life, before the first lumasiran dose, blood oxalate had risen to 32 µmol/L, urine oxalate/creatinine to 573 µmol/mmol and urine glycolate/creatinine to 15 µmol/mmol (Fig. 1A, B). Blood glycolate was 107 µmol/L.

Despite treatment, blood oxalate peaked at 108 µmol/L on day 6, higher than the calcium oxalate saturation limit, roughly corresponding to a blood oxalate concentration of 50 µmol/L. Serum creatinine was normal (0.3 mg/dL).

A gradual decline (65 to 56 to 62 µmol/L at 10, 20 and 30 days, respectively) was observed (Fig. 1A). After the second dose of lumasiran, a further steeper decline of blood oxalate was observed (31 and 17 µmol/L at 45 and 60 days, respectively), reaching the safer upper limit of 12 µmol/L after three doses at 80 days, and the fully normal value of 6 µmol/L from the fourth dose onward (Fig. 1A). However, despite the early start of lumasiran, urine oxalate/creatinine rose to a maximum of 4173 µmol/mmol (normal value for age < 360 µmol/mmol) at 13 days and gradually declined to 765 µmol/mmol at 80 days, after three treatment doses, and to 335 µmol/mmol, the upper level of the normal range after four doses (Fig. 1B), later falling into the normal range at all samplings.

The 24-h urinary excretion of oxalate measured on days 2 and 6 of life was 360 and 400 µmol/day, respectively.

Blood and urine glycolate initially paralleled oxalate, then increased after each lumasiran dose until the third dose, gradually declining afterward (Figs. 1A, B).

Kidney ultrasound was normal at birth and showed only minimal and hyperechogenic spots during the first 2 months. Over 24 months of follow-up, serial ultrasound showed a complete absence of kidney oxalate deposits, and the kidney function remained normal (serum creatinine 0.2 mg/dL) as well as physiological development and growth.

No adverse events were reported.

## Discussion

This is the first report of blood and urine oxalate and glycolate serial analyses in a case of primary hyperoxaluria type 1 treated with lumasiran immediately in the first hours after birth, due to the potentially severe primary hyperoxaluria type 1 prognosis. The biochemical parameters after birth demonstrated normal in utero oxalate clearance, followed by a rapid and steep generation of oxalate in the first hours of life.

Although glycolate oxidase inhibition was started immediately after birth in the absence of previous deposits, it showed a latency of at least 15 days, as demonstrated by the rapidly increasing levels of endogenous oxalate production in the first 7 days. During these days, extremely high levels of urine and blood oxalate, implying calcium oxalate supersaturation, were reached and explained by the physiological perinatal low glomerular filtration rate, notwithstanding hyperhydration, vitamin-B6, and citrate supplementation, thus confirming the potentially aggressive behavior of primary hyperoxaluria type 1, as in his older brother.

It is noteworthy that, despite extremely precocious treatment, blood and urine oxalate required over 3 months to reach normal values, a critical lag of time sufficient to develop irreversible nephrocalcinosis in the most severe forms of primary hyperoxaluria type 1, in spite of aggressive treatment.

The increase in blood glycolate following the administration of lumasiran proved the drug’s action, which led to glycolate accumulation (blood-glycolate 1732 µmol/L, 200-times higher than normal) without appreciable toxic effects, as already reported [16], nor metabolic acidosis or modification of biochemistry parameters.

These observations align with previous data from clinical trials in infants and young children on latency for the lumasiran effect [13, 14]. However, this study represents the first assessment of the metabolism of oxalate and glycolate in a primary hyperoxaluria type 1 patient treated immediately after birth, and provides information on newly generated oxalate, without the influence of oxalate deposit mobilization.

Prior to our study, evidence on early treatment was limited to a small case series which described the treatment outcomes in four infants who received lumasiran before 1 year of age [15]. In addition, the phase III study ILLUMINATE-B included 2 children < 1 year of age at treatment initiation. In these studies, lumasiran was effective without side effects. However, the early treatment did not completely prevent the onset of nephrocalcinosis, which was reported at 2 months of age in the patient with the most severe form of primary hyperoxaluria type 1, who received lumasiran starting from day 9 [15]. Consequently, the Authors hypothesized that higher doses than those proposed in the SmPC might be required because of hepatic immaturity in this patient setting [15].

In our patient, early treatment with lumasiran ensured the absence of nephrocalcinosis for up to 24 months, and, already after 30 days of treatment, blood calcium oxalate saturation became consistently lower than critical limits and normalized after 3 months, without adverse events.

Preemptive treatment in the perinatal period, although questionable since we did not wait to evaluate B6 response, was able to dramatically change the future of this child, for whom a severe form of primary hyperoxaluria type 1 could be predicted, and to avoid disease onset.

Familial neonatal forms of aggressive primary hyperoxaluria type 1 are not comparable to later onset diseases and this innovative and disease-modifying treatment warrants consideration.

## Supplementary Information

Below is the link to the electronic supplementary material.

Supplementary file1 (DOCX 14 KB)

## Declarations

### Availability of data

Clinical data are available upon detailed request.
