# Supplementary material for: Lumasiran at birth changes the trajectory of primary hyperoxaluria type 1: same disease, different outcomes in two affected siblings
Source: J Nephrol. 2025 Jul 9;38(8):2417–22. doi: 10.1007/s40620-025-02325-2 (PMC12630153; doi:10.1007/s40620-025-02325-2)
Supplement: Supplementary file 1 — Supplementary file1 (DOCX 14 KB) [file 40620_2025_2325_MOESM1_ESM.docx]

**Pediatric Oxal Turin Working Group**

Licia Peruzzi, MD, PhD; Vitor Hugo Martins, MSc; Roberta Camilla, MD; Francesca Mattozzi, MD; Bruno Gianoglio, MD; Pediatric Nephrology Unit, Regina Margherita Children’s Hospital, AOU Città della Salute e della Scienza di Torino, Turin, Italy

Luca Marozio, MD, PhD; Obstetrics and Gynecology, Sant’Anna Hospital, AOU Città della Salute e della Scienza di Torino, University of Turin, Italy

Alessandra Coscia, MD, PhD; Neonatal Intensive Care Unit, AOU Città della Salute e della Scienza di Torino, University of Turin, Italy

Silvia Deaglio, MD, PhD; Immunogenetics and Transplant Biology, AOU Città della Salute e della Scienza di Torino Turin, Italy

Marta Leporati, MS; Michele Petrarulo, MS; Domenico Cosseddu, MD; Analytical Chemistry and Kidney Stone Disease Unit, Laboratory of Analysis, AO Ordine Mauriziano di Torino, Turin, Italy
